# Effects of the Spirometry Learning Module on the knowledge, confidence, and experience of spirometry operators

**DOI:** 10.1038/s41533-019-0143-9

**Published:** 2019-08-09

**Authors:** Richard Parsons, David Schembri, Kerry Hancock, Anne Lonergan, Christopher Barton, Tjard Schermer, Alan Crockett, Peter Frith, Tanja Effing

**Affiliations:** 1 Department of Respiratory Medicine, Southern Adelaide Local Health Network, Adelaide, SA Australia; 2 Chandlers Hill Surgery, Happy Valley, SA Australia; 3 Intermediate Care Services, Noarlunga GP Plus, Noarlunga, SA Australia; 4 Department of General Practice, School of Primary and Allied Health Care, Faculty of Medicine, Nursing and Health Sciences, Monash University, Clayton, VIC Australia; 5 Department of Primary and Community Care, Radboud Institute for Health Sciences, Radboud University Medical Center, Nijmegen, The Netherlands; 6 Alliance for Research in Exercise, Nutrition and Activity (ARENA), School of Health Sciences & Sansom Institute for Health Research, University of South Australia, Adelaide, SA Australia; 7 College of Medicine & Public Health, Flinders University, Adelaide, SA Australia

**Keywords:** Physical examination, Asthma

## Abstract

Our study measures effects of the Spirometry Learning Module (SLM) on health-care professionals’ knowledge of spirometry test quality and perceived confidence, experience, and understanding of spirometry measurements and interpretation. Professionals from both primary and hospital-based settings enrolled in the SLM, a training model focusing on spirometry test performance and interpretation, including an online interactive learning component and a face-to-face workshop. Participants were asked to submit patient spirometry assessment worksheets for feedback on quality and interpretation. Data were collected at baseline, SLM completion (20 weeks), and 12 months after SLM completion. Knowledge of spirometry test quality was evaluated with questions relating to five case-based assessments of common spirometric patterns. Perceived confidence, experience, and knowledge in test performance were measured using a 7-point Likert scale. The Friedman test combined with post hoc analyses were used to analyse differences between baseline, 20-week, and 12-month post completion. Qualitative interviews were performed to assess reasons for non-completion. Of the 90 participants enrolled in the SLM and consented to research, 48 completed the 20-week measurement and 11 completed the 12-month measurement. Statistically significant improvements were detected in all outcomes in participants who completed the SLM to 20-week and 12-month follow-up assessments (all *p* values < 0.01). Barriers to completion were limited access to patients requiring spirometry, high clinic workload, and having a different spirometer at the workplace compared to the one used during SLM demonstrations. Our data suggest that participants’ confidence, experience, and knowledge regarding spirometry may improve through SLM completion.

## Introduction

Spirometry is recommended as an indispensable tool in diagnosing, differentiating, and monitoring chronic airways diseases^1–7^ with considerable impact on the accuracy of diagnosis^8,9^ and clinical management of patients with chronic obstructive pulmonary disease (COPD) and asthma in primary care.^8,10^

Although several studies have demonstrated that spirometry can be incorporated into general practices with acceptable levels of technical adequacy, validity, and test quality,^10–12^ the quality of spirometry in primary care is often unacceptably poor.^11–13^ National surveys of Australian and Spanish general practices^14,15^ have both highlighted the need to increase the number of spirometry tests being performed, improve training and knowledge, and improve quality assurance practices, specifically spirometer calibration and maintenance to meet acceptable standards.

Proven strategies to improve the quality of spirometry in primary care include spirometry workshops^12,16^ and incorporation of written and practical spirometry assessments with feedback to practitioners.^12^ Other approaches include open access spirometry clinics^8^ and increased quality assurance initiatives.^11,12^ Factors shown to improve spirometry test quality include the quality and duration of spirometry training,^12,13^ ongoing supervision of operators after completion of training,^13^ use of spirometers that display flow volume curves,^17^ emphasis on end-of-test criteria,^12,18,19^ and allowing time within the daily routine of the practice to perform spirometry.^18^ Several studies have highlighted the need for a more cohesive approach between general practice staff and appropriately trained respiratory professionals.^20^ Close interaction with, and strict technical support by, specialist centres may be the optimal way to provide quality spirometry in general practice.^20–22^

Electronic media (e-learning and e-resources) have been employed in conjunction with online review and automated feedback of test results.^23,24^ More recently, telemedicine-based monitoring systems have shown a positive impact on the quality of spirometry testing.^25^ Approaches directed towards remote support for spirometry interpretation have, however, achieved mixed outcomes. These approaches have included engagement of respiratory specialists to provide a consultative service for interpretation of spirometry measurements^13^ and expert support for spirometry interpretation.^26^

Since spirometry is now firmly established as an essential diagnostic and monitoring tool for chronic respiratory diseases, notably asthma and COPD, it has become increasingly important to promote confidence in its use and ensure high-quality spirometry testing. To this end, we have developed and implemented a spirometry training approach, the Spirometry Learning Module (SLM). In this paper, we measure the effects of the SLM on health-care professionals’ knowledge of spirometry test quality and perceived confidence, experience, and understanding of spirometry measurements and interpretation.

## Results

### Participants

A total of 109 participants, predominantly nurses, enrolled in the SLM, of whom 90 consented to research (Fig. 2). At baseline, 53% of the participants reported that they had performed >5 spirometry tests in the previous 6 months and 50% of the participants reported to have undertaken previous spirometry training (Table 1). Of the 90 participants who commenced the SLM and consented to research, 48 (53%) completed the module and the 20-week post-assessment. At baseline, completers reported a significantly higher perceived knowledge score regarding spirometry measurement and interpretation than non-completers (*p* < 0.05). In addition, significantly more completers reported to have previously undertaken spirometry training (*p* < 0.05) (Table 1). The 12-month assessment was completed by 11 participants (12%).

**Fig. 1:**
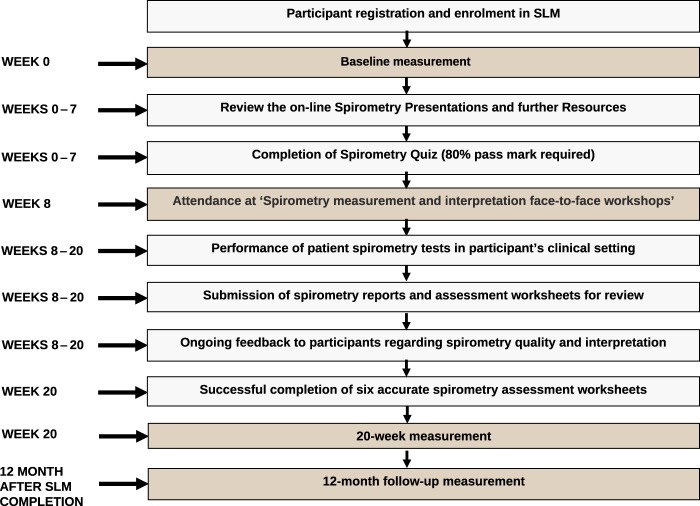
Spirometry Learning Module: Study design flow chart

**Fig. 2:**
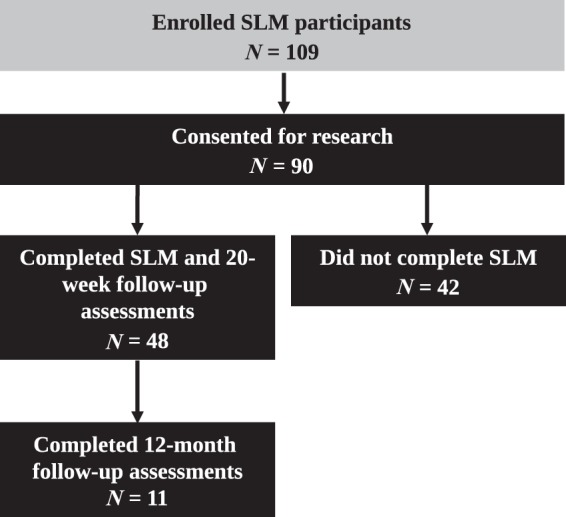
Flowchart of Spirometry Learning Module participants

**Table 1:** Baseline characteristics of participants

Characteristic	All participants, *n* = 90	Completers, *n* = 48	Non-completers, *n* = 42
Knowledge score (median (IQR))	57.1 (31.0–71.4)	60.7 (33.3–72.0)	50.0 (10.7–70.2)
Perceived confidence in spirometry measurement and interpretation (median (IQR))^a^	4 (2–5)	4 (2–5)	4 (2–5)
Perceived experience in spirometry measurement and interpretation (median (IQR))^a^	3 (2–4)	3 (2–5)	3 (1–4)
Perceived understanding of spirometry measurement and interpretation (median (IQR))^a^	2 (1–3)	3 (2–4)	2 (1–3)^b^
Number of people with >5 tests over 6 months (%)	48 (53.4)	30 (62.6)	18 (42.8)
Number of people with previous spirometry training (%)	45 (50.0)	32 (66.7)	13 (31.0)^b^
Current role			
Hospital nurse	37 (41.1)	22 (45.8)	15 (35.7)
Practice nurse	28 (31.1)	13 (27.1)	15 (35.7)
Other^c^	24 (26.7)	12 (25.0)	12 (28.6)

*IQR* interquartile range

^a^The participants’ perceived confidence, experience, and understanding of spirometry measurement and interpretation was assessed using a 7-point Likert scale: 0 = no confidence/experience/knowledge to 7 = very confident/experienced/knowledgeable

^b^Significant (*p* < 0.05) difference between completers and non-completers

^c^Research nurse (*n* = 1); community nurse (*n* = 1); clinical research coordinator/RN (*n* = 1); respiratory nurse in community health setting (*n* = 1); respiratory clinic in a community hospital (*n* = 1); Aboriginal clinical health worker (*n* = 1); clinical nurse in outpatient and inpatient allergy specialty (*n* = 1); pulmonary rehab coordinator (*n* = 1); acting respiratory nurse (*n* = 1); casual registered nurse (*n* = 1); allied health professional (*n* = 1); general practitioner (*n* = 1)

### Baseline and follow-up (FU) assessments

**Fig. 3:**
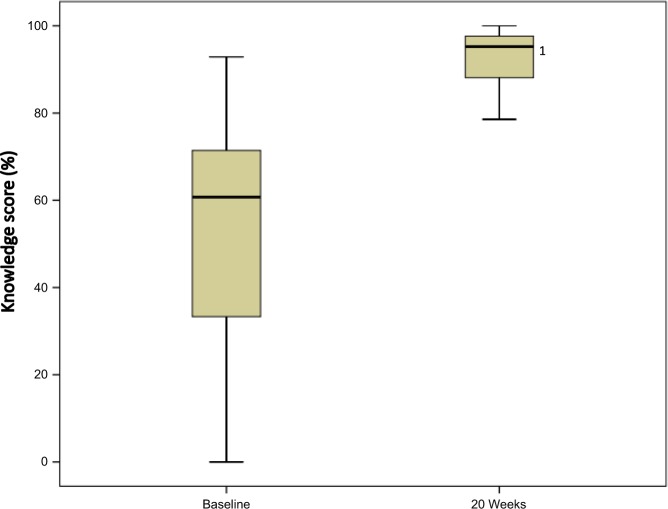
Knowledge scores (%) of completers at baseline and 20 weeks. American Thoracic Society/European Respiratory Society criteria and interpretative strategies were applied to the spirometric data and flow volume curves of 5 common spirometric patterns, 8 questions relating to each of the 5 patterns were posed (40 questions in total); the number of correct answers was expressed as a percentage of the total score. Boxplot: thick line in the middle is the median, the top and bottom box lines show the first and third quartiles, the box is the interquartile range, and the whiskers show the maximum and minimum values. ^1^Significant difference detected between medians (*p* < 0.01); *n* = 42 (as 6 participants had missing values)

**Fig. 4:**
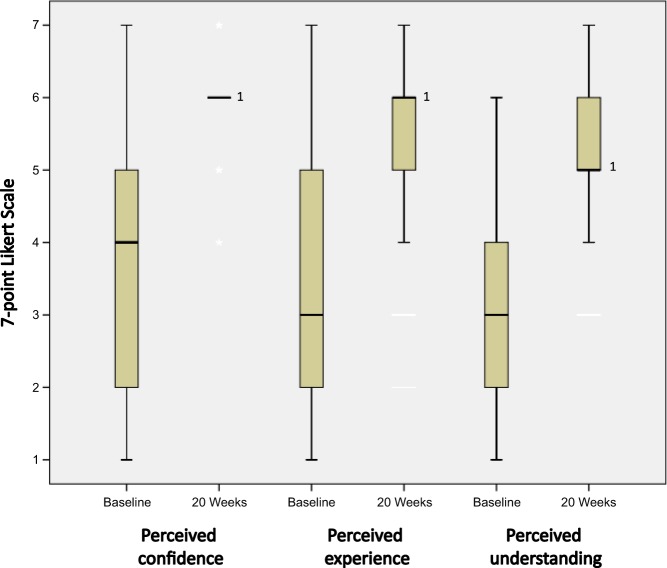
Perceived confidence, experience, and understanding of spirometry measurements and interpretation of completers at baseline and 20 weeks. Boxplot: thick line in the middle is the median (for perceived experience at 20 weeks, the top box line is also the median; for perceived understanding at 20 weeks, the bottom box line is the median), the top and bottom box lines show the first and third quartiles, the box is the interquartile range, and the whiskers show the maximum and minimum values. ^1^Significant difference detected between medians of all three scores (*p* < 0.01); *n* = 45 as three participants had missing values; 7-point Likert scale (0 = no confidence/experience/understanding to 7 = very confident/experienced/knowledgeable)

Baseline and 20-week FU measurements were compared for the 48 completers. The scores all significantly improved for the primary outcome: participants’ knowledge of spirometry measurement quality (baseline 60.7% (interquartile range (IQR) 33.3–72.0%); 20-week FU 94.1% (IQR 88.1–97.6%); *p* value < 0.01; Fig. 3), and for all secondary outcomes (Fig. 4; all *p* values < 0.01).

Outcomes of baseline, 20-week, and 12-month assessments were compared for the 11 participants who completed the 12-month assessment (Table 2). Significant improvements were found in all outcomes over time with all *p* values < 0.01 (Table 2). Post hoc analyses showed significant differences between the baseline assessments and both 20-week and 12-month FU measurements for all outcomes (*p* value < 0.01). No significant differences were found between any of the 20-week and 12-month assessments (Table 2).

**Table 2:** Baseline and follow-up measurements of participants who completed the 12-month assessment (*n* = 11)

Characteristic	Baseline	20 weeks	12 months
Knowledge score (median (IQR))	59.5 (31.0–76.2)	95.2 (90.5–95.2)	90.5 (88.1–95.2)^b^
Perceived confidence in spirometry measurement and interpretation (median (IQR))^a^	4 (2−6)	6 (5−7)	6 (5−7)^b^
Perceived experience in spirometry measurement and interpretation (median (IQR))^a^	3 (2−6)	6 (5−7)	5 (4−7)^b^
Perceived understanding of spirometry measurement and interpretation (median (IQR))^a^	3 (1−4)	5 (5−6)	5 (4−6)^b^

*IQR* inter quartile range

^a^The participants’ perceived confidence, experience, and understanding of spirometry measurement and interpretation was assessed using a 7-point Likert scale: 0 = no confidence/experience/knowledge to 7 = very confident/experienced/knowledgeable

^b^Post hoc tests: significant difference between baseline and 20-week measurement (all *p* values < 0.01) and baseline and 12-month assessment (all *p* values < 0.01); no significant difference between any of the 20-week and 12-month measurements

There was a significant association between the change in knowledge of quality of spirometry measurements between baseline and 20 weeks and the change of perceived confidence in conducting measurements in that same period (*r* = 0.38, *p* = 0.01). This positive association can be classified as medium.^27^

### Reasons for non-completion of the SLM

**Fig. 5:**
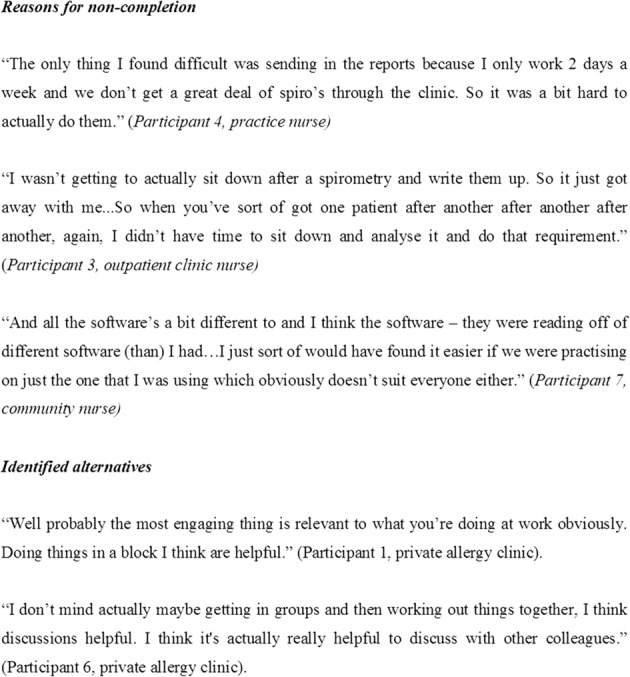
Quotes of non-completers

A total of 42 non-completers were eligible for FU and were contacted by email. Eleven initially agreed to take part in the qualitative interview of whom nine provided phone contact details. The primary barriers to completion of the SLM were described as: limited access to patients considered to require spirometry during the training period (*n* = 5), high clinic workload (*n* = 4), and differences in the type of spirometer used in the workplace to that used during the SLM demonstrations (*n* = 4). Less common barriers were technical/software issues (*n* = 2), too long a period from doing the training to undertaking workplace spirometry (*n* = 2), changed role (*n* = 1), insufficient confidence and knowledge about interpretation of data (*n* = 1), delay in feedback (*n* = 1), and competing professional development demands (*n* = 1) (for participants’ quotes, see Fig. 5). These non-completers identified alternatives that might have supported themselves or others to complete the programme. Most suggested completing all programme aspects in a single day block, perhaps as part of a further FU session (*n* = 7), completing the programme requirements with a colleague (*n* = 5), and tailoring the training for the individual general practice (*n* = 3) (for participants’ quotes, see Fig. 5).

## Discussion

The SLM approach, combining online learning with interactive resources supported by a face-to-face workshop and an integrated approach with respiratory scientists, proved to be effective in participants who successfully completed the SLM. In completers, a significant improvement in knowledge of spirometry test quality was detected at 20 weeks, together with improvements in perceived confidence and experience of participants in spirometry measurement and perceived understanding of spirometry measurement and interpretation. These positive effects persisted in participants who could be followed up to 12 months (*n* = 11). Completers had a significantly higher perceived knowledge score regarding spirometry measurement, better interpretation skills, and were more likely to have previously undertaken spirometry training when compared to non-completers at baseline.

A comparable study to ours, reported after our study was underway, showed improvement in knowledge using case-based assessments.^21^ A mentorship-based intervention was used that involved physicians and allied health team members incorporating an online portal, email, telephone, videoconference, fax, and in-person support.^21^ Whereas the authors concluded that their intervention enhanced knowledge, quality, and actual use of spirometry, long-term feasibility and sustainability of this intervention has yet to be assessed. In contrast, our paper describes the evaluation of an approach that has already been routinely implemented for clinical staff working in both primary care and hospital ward settings.

We were unable to find other studies in the literature that require the submission of a certain number of accurate tests by participants as part of spirometry training. The position statement ‘Spirometry training courses: Content, delivery and assessment’ published in 2017 by the Australian and New Zealand Society of Respiratory Science^28^ mandates the submission of ten tests that have been performed in the workplace for expert review. In our training model, six accurate self-assessments were required for successful course completion and achievement of competency, although only half of all SLM participants were able to submit this number. This may be linked to limited numbers of patients undergoing spirometry testing in the workplace, which may restrict training opportunities and subsequently influence test quality. Future strategies for increasing the number of spirometry test submissions as part of the required evaluation of competency should therefore be developed.

Currently in Australia, spirometry is not controlled under any medical services regulations and as a result it is not mandated for health professionals conducting spirometry testing to achieve recognised competency. As there is no pre-requisite to participate in, much less complete, spirometry training, this may, in part, be responsible for the low number of SLM completers in our study. It may be that participants who completed only the online and face-to-face components did not consider it necessary to continue with the competency assessment.

A positive trend was detected towards successful SLM completion for those participants who had both undertaken previous spirometry training and routinely conducted a higher number of patient spirometry tests in their individual clinical settings. This finding has also been described previously.^12^

While we feel that the perceived increase in participants’ knowledge of spirometry testing and interpretation may translate to an increase in the quality of the spirometry measurements, this was not assessed as a secondary outcome and we have found no compelling evidence for this association in literature. Respondents to a questionnaire circulated as part of a study in Welsh general practices^29^ reported performing spirometry more often if they were confident in conducting testing and this confidence was reflected in the time spent in training. While we assume that an increase in operator confidence will reflect in greater numbers and improved quality of spirometry measurements, this was also not directly measured as an outcome.

Despite our initial thoughts that participants would appreciate less face-to-face contact, our qualitative study results indicate that some participants feel they would benefit from a FU workshop in order to complete the spirometry assessments as a group. The use of telehealth technology, such as webinars, may be employed in subsequent versions of the SLM training to FU with participants after the completion of the practical workshop. Qualitative study data we collected also revealed that participants appreciated the close interaction with the designated respiratory scientist reviewer, an observation already highlighted in previous studies.^20,22,25,30–32^ Participants suggested engaging colleagues to exchange spirometry reports, share ideas, and work through more complex reports may better support programme completion. It is likely that participants who conduct only small numbers of patient spirometry tests find it difficult to have the key concepts reinforced sufficiently to consistently achieve spirometry test quality that meet American Thoracic Society/European Respiratory Society (ATS/ERS) standards. We feel online, case-based practice examples may help with this and we have the intention to include these in subsequent SLM versions.

The main strength of the study was the assessment of spirometry test quality by the participants themselves; as far as we know, previous studies have not used this form of self-assessment confirmed through respiratory scientist feedback. Periodic submission of these assessment worksheets (see Supplementary Fig. 1) allowed previous feedback to be incorporated into later assessments by the participants. This iterative learning approach presented an opportunity to reinforce the practical application of the central concepts over time, allowed the critical criteria to be applied in a standardised manner, and importantly, enabled evaluation of the practical outcomes of the learning approach. This also allowed uniformity in evaluation of submitted assessment worksheets across multiple respiratory scientist reviewers. Standardising the assessment allowed the reviewer to assess not only the participant’s knowledge of the key ATS/ERS acceptability and repeatability criteria but also their practical application. The rapid, online feedback provided a continuous learning platform that could be readily accessed at any time and, more importantly, at times that suited participants. Participants were encouraged to re-access the online resources as often as required both after the face-to-face session and in particular during the review period. Another strength of our study is that, despite non-completion, the qualitative data revealed that satisfaction with all elements of the SLM was very high. Several non-completers reported the SLM resources and subsequent knowledge gained to be valuable and useful in their workplace.

Our study has several limitations. Whereas this pragmatic study showed a large effect on all study outcomes in the group who completed the SLM, only 53% of the participants who commenced the SLM and consented to research completed it. As this study was part of a already implemented training model, participants were not compelled to participate in the research component. Consequently, the FU data of non-completers was not collected and only ‘per-protocol analyses’ were performed. This prevented us from detecting any improvements in confidence and knowledge in participants who had only completed the online and face-to-face components. Although non-completers did not specifically express difficulties in submitting their worksheets (see Supplementary Fig. 1), apart from ‘time constraints’, it is possible that submission of the spirometry reports with the accompanying assessments using scanning and faxing was more onerous than spirometry test reports being automatically uploaded to reviewers as described in other studies.^22,24,33,34^ In addition, since we did not assess the quality of spirometry tests before and after the intervention, we are unable to directly link improvements in confidence and knowledge to actual improved test quality. As the SLM training was conducted in a real-world setting and participants were only invited to participate in the research component when they commenced the online training, participants were not asked to submit test reports for assessment prior to the commencement of the training programme. While there is currently no standardised strategy for assessing spirometry test quality, we feel that the Spirometry Assessment Worksheet (see Supplementary Fig. 1) itself may provide a standardised approach to the assessment of test quality and this should be evaluated in our future studies. Another significant limitation is that a control group not undergoing the SLM was not included as part of this study, consequently the effects cannot formally be attributed to the SLM. Therefore, the effects of this study still need to be confirmed in a randomised controlled trial, which will provide the opportunity to effectively evaluate changes in test quality.

Our results showed that the use of online resources with a practical, face-to-face workshop and submission of a spirometry self-assessment tool may improve confidence, experience, and knowledge regarding spirometry in participants who complete all phases of the training. This approach allows the standardisation of both the learning content and the review and feedback process and evaluates the practical outcomes of the training undertaken. We feel that the use of simple, inexpensive, and interactive tools such as those employed by the SLM could provide a condensed, cost-effective, and sustainable approach to point-of-care spirometry training. The approach could be suitable for incorporating into routine professional development for both primary care medical and nursing practitioners, hospital- or clinic-based nurses, and other health-care providers.

## Methods

### Design and ethics

We have used a pragmatic quasi experimental study design to assess the effects of the SLM on health-care professionals’ knowledge of spirometry test quality and perceived confidence, experience, and understanding of spirometry measurements and interpretation.

This study was approved by the Southern Adelaide Clinical Human Research Ethics Committee (315.13) and we have complied with all relevant ethical regulations. After initiation of the SLM, participants were asked to consent to their SLM data being used for research purposes via the SLM online resources. This consent process was accepted as part of the study protocol approved by the Southern Adelaide Clinical Human Research Ethics Committee.

### Participants

Medical and nursing health professionals from both general practices and health services in regional and metropolitan South Australia and hospital-based settings from the Southern Adelaide Local Health Network could enrol in the SLM.

Participants were able to enrol for the SLM if they were (1) registered as a health professional, (2) conducting spirometry in their clinical setting as part of usual patient care, (3) undertaking patient testing on a spirometer that met ATS/ERS criteria for accuracy and precision and displayed and printed both flow volume curves and volume time graphics, and (4) prepared to commit to all phases of the SLM programme. Only participants who consented to the use of their de-identified responses for research analyses and had successfully completed the SLM by submission of six accurate spirometry assessments were invited to undertake FU measurements at 20 weeks and 12 months by email (these participants were defined as ‘completers’). Regardless of their research consent status, the SLM content and training approach remained identical for all participants.

### Intervention

The SLM employs a ‘blended learning’ approach,^35^ combining online learning and practical face-to-face sessions facilitated by respiratory scientists. This approach is supported by the use of a spirometry self-assessment tool incorporating ongoing feedback and evaluation of operator competency by a respiratory scientist reviewer for 3 months after the face-to-face session.

The SLM training, referred to below as ‘the intervention’, consists of three narrated PowerPoint presentations, a face-to-face workshop directed towards practical application and interpretation of spirometry, and ongoing feedback to participants on the quality and interpretation of their spirometry measurements. The intervention elements and evaluation time points are shown in Fig. 1. The intervention is summarised below, and a more detailed description is available in Supplementary Information. After viewing all three presentations, participants completed an online spirometry quiz to verify their understanding of the fundamental spirometry concepts. These concepts were then practically applied in a 4-h face-to-face workshop facilitated by a senior respiratory scientist. This workshop comprised of two, 2-h sessions. The first session reinforced the practical aspects of spirometry technique, test performance, and measurement, as detailed in the online supplement. The second covered spirometry interpretation and employed a case-orientated approach to reinforce the key indices and interpretative strategies outlined in the online resources.

After the face-to-face workshop, participants were expected to undertake spirometry testing on patients in their individual clinical setting and to complete a Spirometry Assessment Worksheet (see Supplementary Fig. 1) for each patient spirometry test. These were submitted by email or fax to a senior respiratory scientist for review. A corresponding spirometry feedback worksheet (see Supplementary Fig. 2) was then completed by the scientist and returned to the participant. Participants could contact the respiratory scientist at any time for advice during this period. Participants were asked to submit a minimum of two individual patient spirometry tests every 2 weeks in the 12-week review period. They received reminder emails if they failed to submit the quota of patient tests in the required time frame.

### Outcomes and measurements

The primary study outcome was defined as an improvement in participants’ knowledge of spirometry test quality. Secondary outcomes included self-perceived confidence and experience of participants in conducting spirometry, and self-perceived understanding of spirometry measurement and interpretation. Evaluation assessments were performed at baseline, at completion of the SLM (20 weeks), and 12 months after completion of the SLM.

In order to assess participants’ knowledge on the quality of spirometry measurements, a series of case-based assessments of five common spirometric patterns was used. Participants applied the relevant ATS/ERS acceptability and repeatability criteria and interpretative strategies to the test data/results.^36,37^ The assessment included 8 questions relating to each of the 5 cases, 40 questions in total, and the number of correct answers was expressed as a percentage of the total score.

A 7-point Likert scale was used to assess participants’ perceived confidence and experience in conducting spirometry and perceived understanding of spirometry measurement and interpretation (0 = no confidence/experience/knowledge; 7 = very confident/experienced/knowledgeable).

The following information was collected at baseline as it was considered that these variables could be associated with successful completion of the intervention: the number of spirometry tests undertaken in the past 6 months, previous spirometry training, current role, regular place of work, whether the participant was the sole spirometry operator, and details of the spirometer type and model used routinely.

### In-depth interviews with SLM non-completers

Post hoc, we decided to assess reasons for non-completion of the SLM. We therefore conducted in-depth, one-on-one qualitative interviews with participants who consented to further FU research but who did not successfully submit six accurate assessments and subsequently did not achieve competency. The in-depth interviews followed a semi-structured interview guide and were conducted by a researcher experienced in qualitative interviewing who was not involved in the SLM training (C.B.). All interviews were conducted by phone and were audio recorded with permission and then transcribed verbatim. The interview transcripts were imported to NVivo (QSR International, Melbourne, Victoria) to assist with analysis. All transcripts were read in full by two authors (C.B., T.E.). A preliminary coding scheme was developed by one of the authors (C.B.), and a second author (T.E.) confirmed that it incorporated all emerging themes. Then four authors reviewed the coding in detail to confirm the coding of text within each of the categories and themes that emerged. Any disputes were resolved by discussion. Ethics approval from the Southern Adelaide Clinical Human Research Ethics Committee was obtained for this sub-study (315.13).

### Statistical analyses

Baseline characteristics were reported using descriptive analyses. Differences between the primary outcome at baseline, 20 weeks, and 12 months in ‘knowledge of quality of spirometry measurements’ were analysed using Friedman test, followed by post hoc analyses as required. The same analytic approaches were undertaken to assess the secondary outcomes. In addition, associations between the ‘change in the primary outcome from baseline to 20 weeks’ and ‘the change in perceived confidence in conducting measurement in that same period’ were explored using Spearman test. A *p* value < 0.05 was considered to be statistically significant.

### Reporting summary

Further information on research design is available in the Nature Research Reporting Summary linked to this article.

## Supplementary information

Supplementary Information

Reporting Summary

## Data Availability

The data that support the findings of this study are available from the corresponding author, R.P., upon reasonable request.

## Supplementary information

**Supplementary Information** accompanies the paper on the *npj Primary Care Respiratory Medicine* website (10.1038/s41533-019-0143-9).
