# Supplementary material for: Effects of the Spirometry Learning Module on the knowledge, confidence, and experience of spirometry operators
Source: NPJ Prim Care Respir Med. 2019 Aug 9;29:30. doi: 10.1038/s41533-019-0143-9 (PMC6689054; doi:10.1038/s41533-019-0143-9)
Supplement: Supplementary file 1 — Supplementary Information [file 41533_2019_143_MOESM1_ESM.pdf]

# Supplementary Information

**Effects of the Spirometry Learning Module on the knowledge, confidence, and experience of spirometry operators**

**Content:** **Page**


| Content: | Page |
|---|---|
| Extra information regarding intervention | 2 |
| Spirometry Assessment Worksheet | 3 |
| Additional information regarding Assessment Worksheet | 4 |
| Spirometry Feedback Worksheet | 5 |
| References | 7 |

## Extra information regarding intervention

The SLM resides on MOODLE (Modular Object-Oriented Dynamic Learning Environment) open source software Learning Management System https://docs.moodle.org/31/en/AboutMoodle) in which all assessments and questionnaires were integrated. Progression within the SLM was dependent on successful activity completion of previous sections. The key resources consisted of three narrated PowerPoint presentations each of 25 - 30 minutes duration. These presentations included an introduction to spirometry, a guide to measurement, and a case-based strategy for spirometry interpretation. Other online elements (e.g. frequently asked questions, scripts for first time operators, links to websites of interest) were included as additional resources but were not considered essential to successful module completion. After viewing all three presentations, participants completed an on-line spirometry quiz to verify their understanding of the fundamental spirometry concepts. These concepts were then practically applied in the two face-to-face sessions facilitated by a senior respiratory scientist. The first was a two-hour spirometry measurement practical performance session to reinforce the practical aspects of spirometry technique, test performance, and measurement. The second was a two-hour spirometry interpretation education session using a case orientated approach to reinforce the key indices, including relevant ATS/ERS acceptability repeatability criteria, and interpretative strategies (1,2) outlined in the on-line resources . A series of case-based assessments of five common spirometric patterns was used: normal (age dependent), reversible, irreversible, restrictive ventilatory pattern and poor test performance.

**SPIROMETRY LEARNING MODULE - SPIROMETRY ASSESSMENT WORKSHEET**

**ALL QUESTIONS MUST BE ANSWERED** *(refer to the SLM presentation 'Interpretation of Spirometry' and the SLM Spirometry Interpretative Guide' if you require help).*
This assessment worksheet should be completed after each patient spirometry measurement and should be submitted to your assigned respiratory scientist reviewer with a de-identified copy of the original spirometry report.
*Patient's name & medical record number should be removed / blacked out; all other demographics should remain visible.*
**The 3 best pre and post-bronchodilator FVC trials (at least) should be presented for review including the percentage (%) change post bronchodilator** *(you may need to print/submit each of these reports individually).*

**DATE:** 00|00|00 **TIME:** 00:00 **OPERATOR:** OPERATOR NAME **PATIENT I.D. (D.O.B.):** 000000

**SECTION 1:** ***All 7 (seven) compulsory questions in this section must be answered correctly (1 mark for each)***

1. **Apply the ATS/ERS *acceptability* criteria to the best performed PRE-bronchodilator flow-volume curve (Tick whichever is applicable; more than one answer may apply):**
   - ☑ Well performed (no issues observed)
   - ☐ Early termination of expiratory flow
   - ☐ Sub-maximal patient effort
   - ☐ Hesitation at the start of forced expiration
   - ☐ Interruption to expiratory flow (e.g. cough, mouthpiece obstruction by the tongue, glottic closure)
   - ☐ Don't know
2. **Apply the ATS/ERS *repeatability* criteria to all PRE-bronchodilator spirometry measurements (regardless of test quality)**
   - Based on ATS/ERS criteria, FEV1 measurements are repeatable: ☑ Yes ☐ No ☐ Don't know
   - Based on ATS/ERS criteria, FVC measurements are repeatable: ☑ Yes ☐ No ☐ Don't know
3. **Apply the ATS/ERS *repeatability* criteria to all POST-bronchodilator spirometry measurements**
   - Based on ATS/ERS criteria, FEV1 measurements are repeatable: ☑ Yes ☐ No ☐ Don't know
   - Based on ATS/ERS criteria, FVC measurements are repeatable: ☑ Yes ☐ No ☐ Don't know
4. **Assess the best performed PRE-bronchodilator flow-volume curve:**
   - ☑ Appears normal *for age* ☐ Appears abnormal *for age* ☐ Poor test performance
   - ☐ Don't know
5. **Assess the best pre-bronchodilator spirometry measurement based on ATS/ERS criteria:**
   - ☑ All measurements are *within* normal limits ☐ Don't know
   - The following measurements are *less than the lower limit of normal*: ☐ FEV1 ☐ FVC ☐ FEV1/FVC
6. **Is there a positive bronchodilator response (reversibility)?** ☐ Yes ☑ No ☐ Don't know
   **If yes;** which measurements show reversibility? ☐ FEV1 ☐ FVC ☐ Both FEV1 and FVC
7. **Technical Feedback Notes:** ..........
   ALL MEASUREMENTS ARE ACCEPTABLE AND REPEATABLE

**SECTION 2:** ***The following 2 (two) questions ask you to identify the spirometric pattern of the best PRE-bronchodilator flow volume curve and interpret the best PRE-bronchodilator spirometry measurements (1 additional mark for each correct answer).***

1. **Identify the spirometric pattern of the best performed PRE-bronchodilator flow volume curve:**
   - ☑ Appears normal *for age* ☐ Airflow limitation/obstructive ventilatory pattern
   - ☐ Restrictive ventilatory pattern ☐ Poor test performance ☐ Don't know
2. **Interpret the best PRE-bronchodilator spirometry measurement based on ATS/ERS criteria:**
   - ☑ Normal spirometry
   - ☐ Airflow limitation ☐ mild ☐ moderate ☐ moderately-severe ☐ severe ☐ very severe
   - ☐ Restrictive ventilatory pattern ☐ mixed obstructive/restrictive ventilatory pattern
   - ☐ Unable to reliably interpret measurement due to poor test performance ☐ Don't know

Spirometry Learning Module – Spirometry Assessment Worksheet ©

**Supplementary Figure 1. Spirometry Assessment Worksheet**

**Additional information regarding Spirometry Assessment Worksheet (Figure 1S)**

This worksheet allowed us to determine the participant's ability to correctly self-assess the spirometry measurements against key ATS/ERS criteria, specifically the acceptability of test performance, including recognition of common patient related performance issues, the repeatability and normality of measurements and assessment of bronchodilator response. These elements are considered essential to ensure accurate and reliable spirometry test results to ATS/ERS standards. In addition, the accuracy of interpretation of the spirometry data against ATS/ERS severity classifications and pattern recognition of the flow volume curve were assessed.

SPIROMETRY ASSESSMENT FEEDBACK

DATE: 00/00/00 TIME: 00:00: OPERATOR: Participant name PATIENT I.D. (D.O.B.): 000000

*This feedback needs to be viewed in conjunction with your original submitted Test Report & Spirometry Assessment Worksheet*

■ = correct answer ■ = your answer. *IF YOUR ANSWER IS CORRECT ONLY THE GREEN BOX WILL BE VISIBLE*

SECTION 1 *All 7 (seven) compulsory questions in this section must be answered correctly (1 mark for each)*

1. **Application of ATS/ERS acceptability criteria to the best performed PRE-bronchodilator flow volume curve**

   * *This is only applied to the best pre-bronchodilator flow volume curve not to all the curves.*

   ■ Well performed (no issues observed)
   ☐ Early termination of expiratory flow
   ☐ Sub-maximal patient effort
   ☐ Hesitation at the start of forced expiration
   ☐ Interruption to expiratory flow (e.g. cough, mouthpiece obstruction by the tongue, glottic closure)
   ☐ Don't know

2. **Application of the ATS/ERS repeatability criteria to all PRE-bronchodilator spirometry measurements (regardless of patient performance or measurement quality)**

   **To be repeatable the best 2 pre-bronchodilator FEV1 and FVC measurements need to be within 150 mls (0.15L) of each other.*

   Based on ATS/ERS criteria, FEV1 measurements are repeatable : ■ Yes ☐ No ☐ Don't Know

   Based on ATS/ERS criteria, FVC measurements are repeatable: ■ Yes ☐ No ☐ Don't Know

3. **Application of the ATS/ERS *repeatability* criteria to all POST-bronchodilator spirometry measurements**

   **To be repeatable the best 2 post-bronchodilator FEV1 and FVC measurements need to be within 150 mls (0.15L) of each other.*

   Based on ATS/ERS criteria, FEV1 measurements are repeatable : ■ Yes ☐ No ☐ Don't Know

   Based on ATS/ERS criteria, FVC measurements are repeatable: ■ Yes ☐ No ☐ Don't Know

4. Assessment of the best performed pre-bronchodilator flow volume curve:

   **You only need to assess the flow volume curve as either normal or abnormal in appearance (hint; you may want to interpret the spirometry measurements first if you are unsure)*

   ■ Appears normal *for age*
   ☐ Appears abnormal *for age*
   ☐ Poor test performance
   ☐ Don't know

5. Assessment of the best pre-bronchodilator spirometry measurement based on ATS/ERS criteria:

   ■ All spirometry measurements are *within* normal limits ☐ Don't know

   * *FEV1, FVC and FEV1/FVC ratio (or FER) values are all greater than the lower limit of normal (LLN)*

   The following spirometry measurements are less than the lower limit of normal (LLN):

   ☐ FEV1 ☐ FVC ☐ FEV1/FVC (FER)

6. **Is there a positive bronchodilator response (reversibility)?** ☐ Yes ■ No ☐ Don't Know

**IF YES;* which measurements show reversibility? ☐ *FEV1* ☐ *FVC* ☐ *Both FEV1 and FVC*

**Reversibility is considered a 12% (or greater) improvement in* _**either**_ *FEV1 or FVC and in addition an increase of at least 200 mls.*

*While an increase in FVC of 12% or greater and in addition an increase of at least 200 mls is not suggestive of asthma it still indicates reversibility and a clinically significant improvement in lung capacity.*

7. **Appropriateness of Technical Feedback Notes:** ■ Yes ☐ No

**Note if* _any_ *measurements fail to meet repeatability criteria or if the patient performance fails to meet* _any_

*of the acceptability criteria, this must be described in the Technical Feedback Notes.*

**Refer to the 'Suggested Technical Feedback Notes' download from the module resources*

**<u>SECTION 2:</u> *The following 2 (two) questions ask you to identify the spirometric pattern of the best PRE-bronchodilator flow-volume curve and interpret the best PRE-bronchodilator spirometry measurements (1 additional mark for each correct answer).**

8. **Identification the spirometric pattern of the <u>best performed PRE-bronchodilator flow volume curve:</u>**

**Refer to the 'Spirometry Interpretation' download from the module resources; (hint; you may want to interpret the spirometry measurements first if you're unsure)*

■ Appears normal *for age*

☐ Airflow limitation/obstructive ventilatory pattern

☐ Restrictive ventilatory pattern ☐ Poor test performance ☐ Don't know

9. **Interpretation of the <u>best PRE-bronchodilator spirometry measurement</u> based on ATS/ERS criteria:**

**Refer to the 'Spirometry Interpretation' download from the module resources*

■ Normal spirometry **FEV1, FVC and FEV1/FVC (FER) are all greater than the LLN*

☐ Airflow limitation ☐ mild ☐ moderate ☐ moderately severe ☐ severe ☐ very severe

**Airflow limitation is only present when the FEV1/FVC ratio (or FER) is less than the LLN and then the degree of airflow limitation is classified by the FEV1 % predicted*

☐ Restrictive ventilatory pattern **FVC is less than the LLN*

☐ Mixed restrictive/obstructive ventilatory pattern

**FEV1, FVC and FEV1/FVC ratio (or FER) are all less than the LLN*

☐ Unable to reliably interpret spirometry data due to poor patient performance ☐ Don't know

**REVIEWED BY:** Reviewer name **DATE:** 00/00/00

**SECTION 1 (Compulsory Questions):** 7 / 7

**SECTION 2 (Interpretative Questions):** 2 / 2



**Supplementary Figure 2. Spirometry Feedback Worksheet**

**Supplementary References**


1. Miller, M. R., Hankinson, J., Brusasco, V., Burgos, F., Casaburi, R. et al. Standardisation of spirometry. European Respiratory Journal 26, 319-338, doi:10.1183/09031936.05.00034805 (2005)

2. Pellegrino, R., Viegi, G., Brusasco, V., Crapo, R. O., Burgos, F. et al. Interpretative strategies for lung function tests. European Respiratory Journal 26, 948-968, doi:10.1183/09031936.05.00035205 (2005).
